# DISTEVAL: a web server for evaluating predicted protein distances

**DOI:** 10.1186/s12859-020-03938-z

**Published:** 2021-01-06

**Authors:** Badri Adhikari, Bikash Shrestha, Matthew Bernardini, Jie Hou, Jamie Lea

**Affiliations:** 1 Department of Computer Science, University of Missouri-St. Louis, 312 Express Scripts Hall, St. Louis, MO USA; 2 Department of Computer Science, Saint Louis University, 217 Ritter Hall, St. Louis, MO USA

**Keywords:** Protein structure prediction, Distance prediction, Inter-residue contact prediction, Inter-residue contact assessment, Distogram

## Abstract

**Background:**

Protein inter-residue contact and distance prediction are two key intermediate steps essential to accurate protein structure prediction. Distance prediction comes in two forms: real-valued distances and ‘binned’ distograms, which are a more finely grained variant of the binary contact prediction problem. The latter has been introduced as a new challenge in the 14th Critical Assessment of Techniques for Protein Structure Prediction (CASP14) 2020 experiment. Despite the recent proliferation of methods for predicting distances, few methods exist for evaluating these predictions. Currently only numerical metrics, which evaluate the entire prediction at once, are used. These give no insight into the structural details of a prediction. For this reason, new methods and tools are needed.

**Results:**

We have developed a web server for evaluating predicted inter-residue distances. Our server, DISTEVAL, accepts predicted contacts, distances, and a true structure as optional inputs to generate informative heatmaps, chord diagrams, and 3D models. All of these outputs facilitate visual and qualitative assessment. The server also evaluates predictions using other metrics such as mean absolute error, root mean squared error, and contact precision.

**Conclusions:**

The visualizations generated by DISTEVAL complement each other and collectively serve as a powerful tool for both quantitative and qualitative assessments of predicted contacts and distances, even in the absence of a true 3D structure.

## Background

The most demonstrated successful methods in the recent 13th and 14th Critical Assessment of Protein Structure Prediction (CASP13 and CASP14) experiments, including AlphaFold [1], have unanimously agreed that accurate contact and distance prediction are the key to furthering progress in the field of protein structure prediction [2–4]. Since these predictions serve as input to the subsequent process of three-dimensional (3D) modeling, it is sensible to assess and evaluate predicted contacts and distances on their own. The quality of the predicted 3D model is dependent on the quality of the predicted contacts or distances, whichever is used as the input. To evaluate predicted contacts, metrics such as Matthew’s correlation coefficient (MCC) and precision are commonly used [5, 6], and web servers such as EVAcon [7] and ConEva [8] have also been developed for this purpose. Distance prediction approaches, on the other hand, are very recent. In May 2020, the organizers of CASP14 introduced a new challenge category—inter-residue distance prediction. Moreover, recent methods such as trRosetta [9] and AlphaFold [1] predict distograms, and others such as PDNET [10] and the Generative Adversarial Network-based methods [11] predict real-valued distances. Despite these advancements, methods for assessing and evaluating predicted distances remain poorly explored. To fill this void we developed DISTEVAL, a web server for assessing and evaluating predicted contact and distances. In addition, a downloadable version of DISTEVAL can be used to perform evaluation through 3D modeling.

## Implementation

Given user supplied contact or distance predictions, DISTEVAL performs both qualitative assessment as well as quantitative evaluation. Qualitative visual assessments are presented as (a) heatmaps, and (b) chord diagrams. When the corresponding true structure is provided, an interactive 3D model visualization complements the assessments. The design of our implementation allows a user to easily draw correlations between the heatmaps, chord diagrams, and the true structure, allowing for deeper insight into the predictions than simple quantitative metrics. For drawing the heatmaps and chord diagrams, users can choose from three coloring schemes - the default gradient based coloring, a coloring scheme that highlights distances around 8 and 12 Å, and a perceptually uniform coloring (easier for color-blind users).

### Heatmaps

**Fig. 1:**
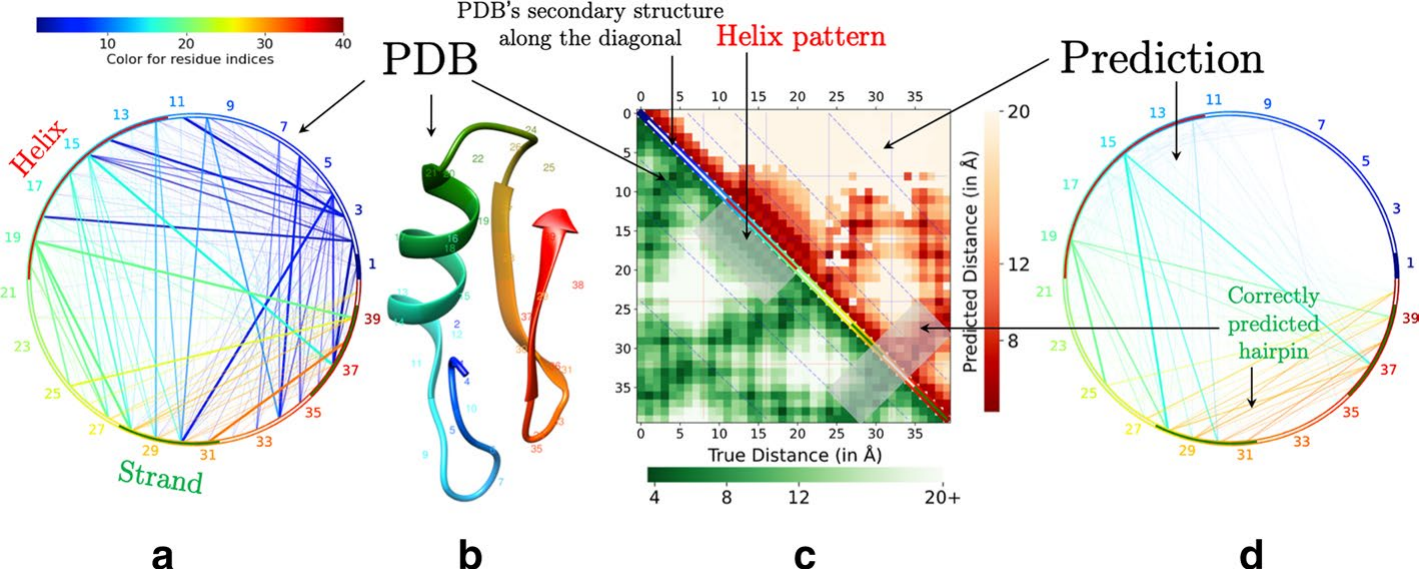
**a** Chord diagram of input PDB structure. The arch of the chord diagram shows the secondary structure labels by DSSP [12] (helix in red, coil in white, and strand in green). Chord widths and transparency correspond to the actual physical distances (smaller distances have larger widths and lesser transparency) and chord colors are based on the residue index number—red through green to blue. **b** The structure of the corresponding PDB file. **c** The heatmap diagram with the predicted contact map shown in the upper triangle with the PDB distance map in the lower triangle. The colors in the diagonal line of the heatmap show the DSSP assigned secondary structures (helix in red, coil in white, and strand in green). **d** Chord diagram of the predicted contact map; Chord widths correspond to the predicted contact confidence. The chord diagram (in **d**) and the heatmap (in **c**) clearly highlight the patterns such as strand-strand interaction

When generating heatmaps of distance predictions, we ignore all true and predicted distances higher than 20 Å and ceil all distances below 3.5 Å to 3.5 Å. This serves an important function. With such a standardization, heatmaps from different structures or predictions, including those from different proteins, can be readily compared as they will have the same numeric range and color mapping. This also allows for a clear visualization that focuses on interactions (shorter physical distances) which are more important than non-interactions [6]. When contact predictions are provided as input, the heatmap coloring corresponds to contact prediction confidence scores. Exploiting the fact that distance and contact maps are symmetrical with respect to the main diagonal line, we reserved the upper triangle for the predicted distance/contact map and the lower triangle for a true distance map. Combining the heatmaps in this way eases comparison between the two. When a native structure is supplied, three-class secondary structure calculations, i.e., helix, strand, and coil, from the Define Secondary Structure of Proteins (DSSP)[12] program, are shown on the main diagonal line of the heatmap as red, green, and white bands. This allows the helix-helix, strand-strand, and helix-strand interactions to be easily studied in a heatmap (see Fig. 1c) when they would otherwise be non-apparent. In the absence of a true structure, the main diagonal is colored with a gradient, associating each residue with a unique color. This provides a correspondence with the chord diagram, allowing the user to synthesize information from the two. When a true structure is provided along with a distance map, a second heatmap is generated showing the absolute error of the prediction for each residue pair (pixel). Since it is easier for the human eye to perform side-to-side (or top-down) comparison, we allow all heatmaps to be rotated $45^{\circ }$ so the diagonal line is vertical. This further eases visual assessment as the main diagonal is now displayed vertically with its associated residue pair distances horizontally oriented.

### Chord diagrams

Chord diagram visualizations can distinctly highlight long-range interactions (short physical distances between long-range residue pairs). By arranging the amino acid sequence in a circle, the sequence length separating two interacting residues is proportional to the chord length representing their interaction. Unlike the heatmaps, however, distance is represented not by color but by the thickness of the chord, i.e. shorter physical distances are thicker (see Fig. 1a). In the case of contacts, the thickness represents prediction confidence. The border arc of the chord diagram is colored similar to the corresponding heatmap’s main diagonal line which allows the user to draw a correspondence between the heatmap and the chord diagram. When a true structure is provided, the secondary structural classes appear. In the absence of true structure, the same color gradient, as in the heatmap’s diagonal line, is applied. For each residue pair i–j, the corresponding chord is colored according to the residue with lower sequence index. This assists the user in finding “interaction hubs” [6], regions where residues commonly interact with residues in another region (as the chords are all similarly colored). More generally this allows the user to see how residues in a particular region interact with the rest of the sequence. The chord coloring remains when the true structure is provided, making it especially useful when paired with 3D models. Two separate chord diagrams are drawn for the prediction and the true structure, which allows a side-by-side comparison of the true and predicted distances/contacts. Missing long-range predictions are especially easy to detect with chord diagram visualizations.

### 3D models

These complementary heatmaps and chord diagrams also enable comparisons of predicted distances or contacts with the true 3D structure. When the native PDB structure is supplied, DISTEVAL also visualizes the structure using JSmol [13] allowing the 3D models, chord diagrams and heatmaps to be studied together. By default, the same gradient is used for coloring heatmaps, chords, and the 3D model. This allows the user to visually connect all produced visualizations, enabling a better understanding of each. Details of a prediction that may be hard to discern in one visualization can easily be traced to the 3D model, while the complexities of the 3D model are unpacked by 2D visualizations. This allows the user to extract information about a prediction’s structural details, which is often missed by quantitative metrics. The user can thus develop a better understanding of the predictions, their relationship to the true structure, and, by extension, the strengths and limitations of the methods that produced them.

### Quantitative evaluation

To quantitatively evaluate predicted distances and contacts, when a true structure is available, DISTEVAL provides numerous metrics including mean absolute error (MAE) of the long-range distances, root mean squared error (RMSE), local distance difference test (LDDT) score [14], and precision of medium and long-range contacts. Following the widely-adopted standard in the field of protein structure prediction [5, 6], we define local, short-range, medium-range, and long-range distances between residue pairs by the sequence separation length interval they fall into: $d_\text {local}< 6 \le \ d_\text {short}< 12 \le d_\text {medium} < 24 \le d_\text {long}$. We also define the top L/5 and top L as the most confident top L/5 or top L predictions where L is the length of the protein sequence or the number of the valid residues in the corresponding native structure, whichever is smallest. Previous studies have shown that long-range contacts, i.e., short distances between pairs separated by at least 23 residues in the sequence, are the most informative pairs for accurate reconstruction [5, 6]. In order to calculate the precision for predicted distances, we translate distance predictions into contacts and evaluate precision for: (a) long-range contacts, and (b) medium and long-range contacts. For a more rigorous assessment of predicted distances, we extended the CONFOLD method [15], so a user can build 3D models from the predicted distances, and compare these reconstructed models with the true structure to obtain TM-score [16] of the top or best model. This TM-score serves as a ‘utility score’ of the predicted distance map. It is important to note that CONFOLD is an appropriate choice for building 3D models because of its speed and non-reliance on any information other than the predicted distance information. Since it typically takes a few minutes to a few hours to execute this distance-guided 3D modeling process, this script is available as a separate tool that can be downloaded and run locally.

**Fig. 2:**
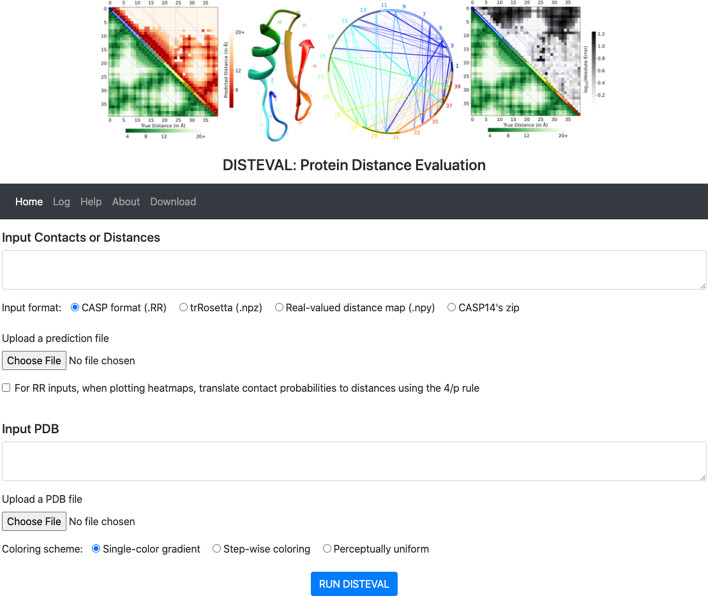
A screenshot of DISTEVAL’s homepage. It accepts contact/distance files and/or a PDB file as input. Input files can be: **a** uploaded, **b** copy-pasted into the textarea, or **c** referenced using a hyperlink

### Technologies used

DISTEVAL was developed using Flask (https://flask.palletsprojects.com), a micro-framework for web application development in Python. Flask uses the Werkzeug WSGI toolkit (https://werkzeug.palletsprojects.com/en/1.0.x/) and the Jinja2 web templating engine (https://jinja.palletsprojects.com/en/2.11.x/) for Python which helps to render dynamic web pages. The routing, debugging, and web server from Werkzeug, together with the templating from Jinja, make Flask a lightweight and easy to use full-stack web application framework. This enabled us to quickly develop and iterate upon DISTEVAL development and set the stage for future improvements to meet the needs of the protein structure prediction community. A Python server was created to host the application. HTTP requests and responses are handled by the WSGI layer which interfaces with several Python scripts we wrote to process client requests and generate the visualizations. When a true structure is provided, the scripts use DSSP to determine secondary structure classes and JSmol to render the 3D models. The outputs from these scripts are returned to the client as an HTTP response, which is shown in the output HTML page.

## Results

As shown in Fig. 2, DISTEVAL accepts predicted contacts and distances in various formats including the CASP13 RR format^1^ and the new CASP14 RR format^2^. Protein structures are accepted as the standard protein data bank (PDB) files. Also accepted are predicted $C\beta$ (carbon-beta) distance maps as Numpy array files, i.e. in ‘.npy’ file format, and trRosetta[9] predictions in ‘.npz’ file format. A CASP14 zip file contains predictions from all participating groups. DISTEVAL visualizes all these predictions in a single place enabling users to compare the different submissions. DISTEVAL accepts contact predictions (RR files) in all three formats: a) CASP13, i.e., the old CASP format, b) CASP14 format with RMODE set to 1, and c) CASP14 format with RMODE set to 2. The following use cases are supported:

1. Distance/contact prediction without a 3D structure
  - Contacts/distance-bins in CASP’s ‘.RR’ formats
  - Real-valued distance map as a Numpy 2D array in ‘.npy’ format
  - Distance predictions by the trRosetta method in ‘.tgz’ format
  - An RR-zip-file from the CASP14 download area^3^
2. A 3D structure ‘.pdb’ file as the only input
3. Distance/contact predictions along with a 3D structure
  - Contacts/distance-bins in CASP’s ‘.RR’ formats
  - Real-valued distance map as a Numpy 2D array in the ‘.npy’ format
  - Distance predictions by the trRosetta method in the ‘.tgz’ format

Since CASP has now standardized distance bin definitions, ‘RMODE 2’ will most likely remain the standard format for distogram-type predictors. In the case of RMODE 2, predicted contacts are extracted in the usual way. Distance bins, however, require special handling. These are translated to distance maps by using the midpoint of the bin with the highest confidence score. This flattening of distogram-type predictions does lose some information (distograms can have multiple peaks) but no method can precisely project the predicted probability information onto a 2D heatmap. This method is particularly useful as it allows a direct comparison with contact maps and real-valued distance maps. Since distance and contact maps are symmetrical about the main diagonal, we only consider the upper triangle of the matrix for all purposes—visualization as well as quantitative evaluation. Users can provide file input in three ways: (a) a file upload from a client’s local computer, (b) raw text content pasted into the text area, and (c) a uniform resource locator (URL) of the files.

We also performed experiments to obtain a measure of effectiveness for various distance evaluation metrics we implemented, including mean absolute error (MAE), root mean squared error (RMSE), local distance difference test (LDDT) of C$\beta$ atoms at standard sequence separation thresholds such a 6, 12, and 24. At first, with the help of the CONFOLD tool [15], we built three-dimensional models for 150 protein chains in the PSICOV dataset using the real-valued distances predicted by the PDNET-Distance method [10]. Next, we evaluated these distance maps using the various distance evaluation metrics and separately evaluated the top-one models using template-modeling score (TM-score) and global distance test total score (GDT-TS) [16]. Finally, we calculated the Pearson correlation coefficients between the 150 TM-score and 150 GDT-TS values with each of the various distance evaluation metrics. Table 1 lists eight of the top ranking metrics along with their Pearson correlation coefficient with TM-score and GDT-TS. Overall, we find that C$\beta$-based LDDT scores are the most effective metrics to evaluate predicted real-valued distances. These results align with the findings that the metrics pairwise distance test (PDT) and high-accuracy pairwise distance test (PHA), which are similar to C$\beta$-LDDT, are effective metrics to evaluate predicted distances [17].

**Table 1:** Pearson correlation coefficient of metrics used for evaluation of predicted real-valued distances with TM-score and GDT-TS score, calculated on the PSICOV 150 protein dataset

Real-valued distance evaluation metric	PCC* with TM-score	PCC* with GDT-TS
Cb-LDDT of all medium and long-range distances	0.83	0.86
Cb-LDDT of all short, medium, and long-range distances	0.82	0.87
Cb-LDDT of all long-range distances	0.81	0.82
PCC* of all medium and long-range distances below 20 Å	0.77	0.79
MAE of all medium and long-range distances below 20 Å	− 0.72	− 0.78
MAE of all long-range distances below 20 Å	− 0.71	− 0.74
RMSE of all medium and long-range distances below 20 Å	− 0.60	− 0.67
RMSE of all long-range distances below 20 Å	− 0.60	− 0.64

*Pearson correlation coefficient

## Conclusions

The assessment of predicted contacts and distances using heatmaps, chord diagrams, and 3D models complement each other. They collectively serve as a powerful tool to compare and assess predicted contacts and distances even in the absence of a true 3D structure. DISTEVAL comes pre-loaded with many examples ready to run, allowing users to quickly and easily begin understanding protein distance and contact predictions in previously unavailable ways.

## Availability and requirements

Project name: DISTEVAL

Project home page: http://deep.cs.umsl.edu/disteval/

Open-source repository: https://github.com/ba-lab/disteval

Operating system(s): Platform independent

Programming language: HTML, CSS, and Python

License: GNU GPL

Any restrictions to use by non-academics: None

## Data Availability

The datasets generated and/or analysed during the current study are available at the web server itself.

## Abbreviations

CASP: Critical assessment of techniques for protein structure prediction experiment
2D: Two dimensional
3D: Three dimensional
MCC: Matthew’s correlation coefficient
PDNET: Protein distance net framework
DSSP: Define secondary structure of proteins
PDB: Protein data bank
MAE: Mean absolute error
RMSE: Root mean squared error
WSGI: Web server gateway interface
HTTP: Hypertext transfer protocol
HTML: Hypertext markup language
RR: CASP residue-residue contact format
URL: Uniform resource locator
CSS: Cascading Style Sheets
TM-score: Template-modeling score
GDT-TS: Global distance test total score
LDDT: Local distance difference test
$C\beta$: Carbon-beta atom
RMODE: Residue-residue contact mode in CASP RR format
PCC: Pearson correlation coefficient
PDT: Pairwise distance test
PHA: High-accuracy pairwise distance test

## Footnotes

1 https://predictioncenter.org/casp13/index.cgi?page=format#RR

2 https://predictioncenter.org/casp14/index.cgi?page=format#RR

3 https://predictioncenter.org/download_area/CASP14/server_predictions/
